# Dynamic Changes in the Systemic Inflammation Response Index Predict the Outcome of Resectable Gastric Cancer Patients

**DOI:** 10.3389/fonc.2021.577043

**Published:** 2021-02-25

**Authors:** Zhenhua Liu, Haijue Ge, Zhilong Miao, Shoupeng Shao, Hongtai Shi, Congsong Dong

**Affiliations:** ^1^ Department of Radiotherapy, The First People’s Hospital of Yancheng, Yancheng, China; ^2^ Department of Gastroenterology, The Third People’s Hospital of Yancheng, Yancheng, China; ^3^ Department of General Surgery, The Third People’s Hospital of Yancheng, Yancheng, China; ^4^ Department of Oncology, The Third People’s Hospital of Yancheng, Yancheng, China; ^5^ Department of Radiotherapy, The Third People’s Hospital of Yancheng, Yancheng, China; ^6^ Department of Radiology, The Third People’s Hospital of Yancheng, Yancheng, China

**Keywords:** systemic inflammation response index, gastric cancer, prognosis, neutrophil/lymphocyte ratio, platelet/lymphocyte ratio, monocyte/lymphocyte ratio

## Abstract

The systemic inflammation response index (SIRI) has been revealed to be closely related to the prognosis of a variety of tumors. Whether the dynamic change in SIRI before and after surgery can be used to judge the prognosis of patients after radical gastrectomy has not yet been studied. In this study, the predictive ability of preoperative SIRI and changes in SIRI before and after surgery for the survival rate of gastric cancer patients was evaluated in two independent cohorts. It was found that SIRI was closely related to TNM staging. The higher the TNM stage, the higher the proportion of patients with a high SIRI. However, SIRI was not related to any other clinicopathological parameters. Kaplan-Meier survival analysis showed that a high SIRI was associated with poor prognosis in gastric cancer patients in the original cohort and in the validation cohort. SIRI, NLR, PLR, and MLR could be used to judge the prognosis of patients with operable gastric cancer. However, multivariate analysis suggested that only SIRI was an independent prognostic factor for patients with operable gastric cancer. In addition, the change in SIRI at 4 to 6 weeks after surgery compared with SIRI before surgery was closely related to the survival of gastric cancer patients. Compared with the unchanged group (absolute variation <50%), gastric cancer patients with a SIRI increase >50% had a worse OS, while patients with a SIRI decrease >50% had a better prognosis. In conclusion, SIRI can be used as a reliable index to evaluate the prognosis of patients with operable gastric cancer, and the dynamic change in SIRI before and after surgery is significantly related to the prognosis of patients with gastric cancer.

## Introduction

Gastric cancer is one of the most common causes of cancer deaths around the world (1), and it is also the second most common cancer and the third major cause of cancer-related death in China (2). Although great progress has been made in the diagnosis and treatment of gastric cancer in recent years, the 5-year survival rate is relatively low due to the local recurrence or metastasis of primary gastric cancer after resection, making it a global burden.

Studies have confirmed that the microenvironment dominated by inflammatory mediators plays key roles in the occurrence and progression of gastric cancer (3, 4). Generally, the individual’s systemic inflammatory state will be reflected in the changes in the relative levels of circulating white blood cells (WBCs) in the peripheral blood, mainly manifested as changes in neutrophils, monocytes, and lymphocytes. However, the individual’s inflammatory response is a complex process involving many cells and molecules. Increasing numbers of studies have found that peripheral blood neutrophils, platelets, WBC counts, lymphocytes, monocytes, platelet/lymphocyte ratio (PLR), neutrophil/lymphocyte ratio (NLR), and monocyte/lymphocyte ratio (MLR) are closely related to the therapeutic effect and prognosis of gastric cancer patients (5–8). Recently, Qi et al. established a novel systemic inflammation response biomarker, the systemic inflammation response index (SIRI), which is based on peripheral neutrophil, monocyte, and lymphocyte counts. It has been confirmed that SIRI can be used to predict the outcomes of pancreatic cancer patients undergoing chemotherapy to help improve the treatment effect (9). Subsequently, SIRI was found to be closely related to the prognostic value of many other tumors, and this finding has been confirmed in multiple studies. Li et al. confirmed that SIRI is a useful prognostic index for gastric cancer patients, and patients in the low SIRI group can benefit significantly from postoperative adjuvant chemotherapy (10). Zhang et al. also found that the SIRI can predict the overall survival (OS) of patients with gastric cancer after surgery, and the prognosis of patients with high SIRI is poor (11). The dynamic changes in SIRI before and after surgery can also be used to evaluate the OS of patients with esophageal and cervical cancer (12, 13). However, the impact of the dynamic changes in SIRI before and after gastric cancer surgery on the prognosis of patients has not been evaluated. This study explored the impact of inflammatory response-related indexes on the prognosis of patients with resectable gastric cancer and evaluated the predictive ability of preoperative SIRI and changes in SIRI before and after surgery for the survival rate of gastric cancer patients in two independent cohorts to guide clinical practice and to improve the clinical outcomes of gastric cancer patients.

## Methods

### Patients

In this study, gastric cancer patients who underwent radical gastrectomy at Yancheng City No. 1 People’s Hospital from 2011 to 2016 were enrolled (n=442) as the primary cohort. In addition, patients with gastric cancer who underwent radical resection (n=152) were enrolled from Yancheng Third People’s Hospital as the validation cohort. The inclusion criteria were as follows. 1) Gastric cancer was diagnosed based on histological evidence and the eighth edition of the AJCC classification. 2) The pathological type of all patients was gastric adenocarcinoma, and other gastric malignancies, such as lymphoma, gastrointestinal stromal tumor, and residual gastric cancer, were excluded from the study. All patients with gastric cancer underwent full or partial gastrectomy and standard lymphadenectomy. 3) There was no antitumor treatment before surgery, such as chemotherapy or radiotherapy. 4) The life expectancy was ≥ 3 months after surgery. 5) None of the patients had blood diseases, infections or a high fever. 6) All patients had routine blood data 1 week before the operation and routine blood data 4–6 weeks after the operation. The original cohort group included 442 patients. The validation cohort included 152 patients. All patients signed informed consent forms. All studies were conducted in accordance with the Helsinki Declaration. This retrospective study was approved by the Ethics Committee of the First People’s Hospital of Yancheng and the Third People’s Hospital of Yancheng.

### Follow-Up

After surgery, each patient was regularly followed up until December 2018 or until death. Routine examinations were carried out at each follow-up. The OS was calculated from the date of the operation to December 2018 or death. The follow-up period of the original cohort ranged from 4 to 68 months, with a median follow-up time of 35 months.

### SIRI

**Figure 1:**
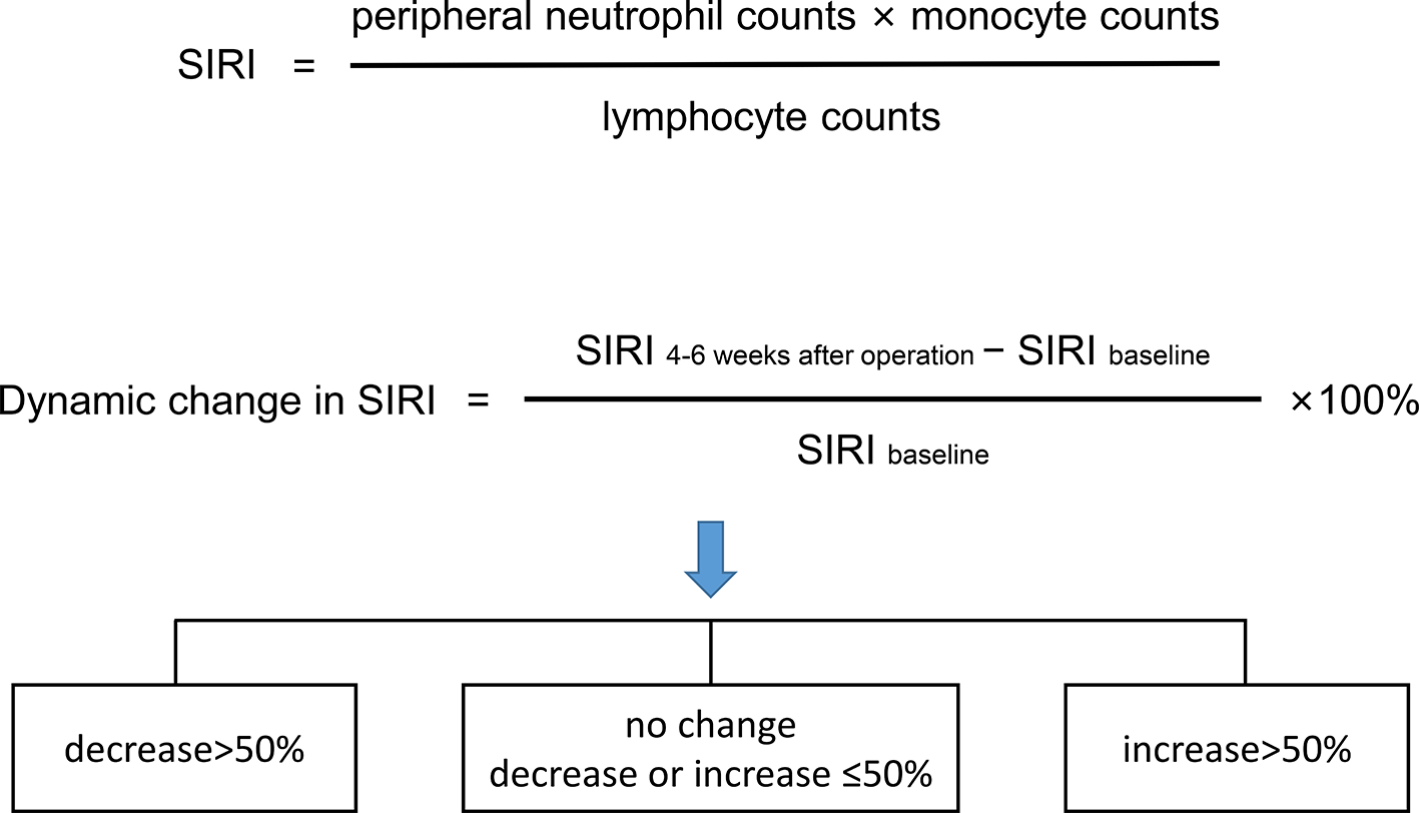
The formula of systemic inflammation response index (SIRI) and dynamic changes in SIRI.

Peripheral venous blood was collected within 3 days before surgery and 4–6 weeks after surgery. The counts of the lymphocytes, neutrophils, monocytes, and platelets in the collected samples were detected using a fully automatic peripheral blood analyzer. NLR, PLR, MLR, and SIRI were detected according to previous formulas (12). Receiver operating characteristic (ROC) curve analysis was used to determine the optimal cut-off values of NLR, PLR, MLR, and SIRI. SIRI within 3 days before the operation was used as the baseline. The dynamic change in SIRI was calculated as a percentage [(SIRI at 4–6 weeks after operation—baseline SIRI)/baseline SIRI×100] and divided into 3 groups [decrease>50%, no change (decrease or increase of no more than 50%), and increase >50%] (**Figure 1**).

### Statistical Analysis

The statistical method used was similar to that used in our previous article (6). SPSS 21.0 software (SPSS Inc., Chicago, IL, USA) was used for the statistical analysis.

## Results

### Clinical Characteristics of the Patients

In the primary cohort group (n=442), there were 295 males (66.7%) and 147 females (33.3%) aged 23–89 years (57 years on average). In terms of the Lauren classification, there were 198 cases (44.8%) of the intestinal type, 73 cases (16.5%) of the diffuse type, and 171 cases (38.7%) of the mixed type. Based on the 8th AJCC stage, 101 (22.9%), 136 (30.8%), and 205 (46.4%) patients were in stages I, II, and III, respectively. Other clinicopathological parameters of the original cohort patients and the validation cohort patients are shown in **Table 1**.

**Table 1:** Clinicopathological characteristics of patients with gastric cancer in primary cohort and validation cohort.

Characteristic	Primary cohort (n=442)		Validation cohort (n=152)	
	No. of patients	%	No. of patients	%
Sex				
Male	295	66.7	115	75.7
Female	147	33.3	37	24.3
Age				
≤60	304	68.8	97	63.8
>60	138	31.2	55	36.2
Tumor location				
Upper	74	16.7	15	9.9
Middle	173	39.1	50	32.9
Lower	195	44.1	87	57.2
Histological grade				
Well or moderately differentiated	205	46.4	91	59.9
Poorly or not differentiated	237	53.6	61	40.1
Lauren type				
Diffuse	73	16.5	33	21.7
Intestinal	198	44.8	65	42.8
Mixed	171	38.7	54	35.5
Tumor size				
≤5	183	41.4	70	46.1
>5	259	58.6	82	53.9
Lymphovascular invasion				
No	323	73.1	91	59.9
Yes	119	26.9	61	40.1
Perineural invasion				
No	258	58.4	102	67.1
Yes	184	41.6	50	32.9
TNM stage (AJCC, 8^th^)				
I	101	22.9	42	27.6
II	136	30.8	77	50.7
III	205	46.4	33	21.7
Adjuvant chemotherapy				
No	177	40.0	57	37.5
Yes	265	60.0	95	62.5

The optimal cut-off value based on the primary cohort was determined by ROC curve analysis as follows: NLR 1.32, PLR 128, MLR 0.24, and SIRI 0.85. Then, the patients were divided into two groups according to the cut-off value. Subsequently, the correlations between SIRI and the clinicopathological characteristics were analyzed in the primary cohort, and it was found that SIRI was closely related to the AJCC stage. The higher the TNM stage was, the higher the proportion of a high SIRI (**Table 2**). SIRI had no statistical relationships with sex, age, tumor location, histological grade, Lauren type, tumor size, lymphovascular invasion, perineural invasion, or adjuvant chemotherapy (**Table 2**). Similar results were obtained in the validation cohort (**Table 2**).

**Table 2:** Baseline characteristics for patients with SIRI ≤ 0.85 *versus* SII>0.85 in primary and validation cohort.

Clinical parameter	Primary cohort				Validation cohort			
	SIRI ≤ 0.85 (214)	SIRI > 0.85 (228)	χ^2^	*P*	SIRI ≤ 0.85 (90)	SIRI > 0.85 (62)	χ^2^	*P*
Sex			0.01	0.972			2.26	0.133
Male	143	152			72	43		
Female	71	76			18	19		
Age			2.18	0.140			0.403	0.699
≤60	140	164			55	42		
>60	74	64			35	20		
Tumor location			0.25	0.884			1.01	0.603
Upper	35	39			10	5		
Middle	82	91			53	34		
Lower	97	98			27	23		
Histological grade			0.03	0.854			0.14	0.706
Well or moderately differentiated	94	111			55	36		
Poorly or not differentiated	120	117			35	26		
Lauren type			1.93	0.382			0.26	0.880
Diffuse	39	34			19	14		
Intestinal	89	109			40	25		
Mixed	86	85			31	23		
Tumor size			0.01	0.907			0.03	0.855
≤5	88	95			42	28		
>5	126	133			48	34		
Lymphovascular invasion			0.12	0.729			0.01	0.968
No	158	165			54	37		
Yes	56	63			36	25		
Perineural invasion			0.96	0.326			0.02	0.890
No	130	128			60	42		
Yes	130	128			30	20		
TNM stage (AJCC, 8^th^)			30.85	<0.001^*^			15.10	0.001^*^
I	72	29			35	7		
II	65	71			41	36		
III	77	128			14	19		
Adjuvant chemotherapy			0.06	0.800			0.18	0.670
No	87	90			35	22		
Yes	127	138			55	40		
NLR			26.363	<0.001^*^			22.12	<0.001^*^
NLR ≤ 1.32	133	86			41	6		
NLR>1.32	81	142			49	56		
PLR			18.194	<0.001^*^			13.984	<0.001^*^
PLR ≤ 128	99	61			61	23		
PLR>128	115	167			29	39		
MLR			25.93	<0.001^*^			42.93	<0.001^*^
MLR ≤ 0.24	140	94			81	25		
MLR>0.24	74	134			9	37		

*represents statistical significance.

### Survival Analysis

**Figure 2:**
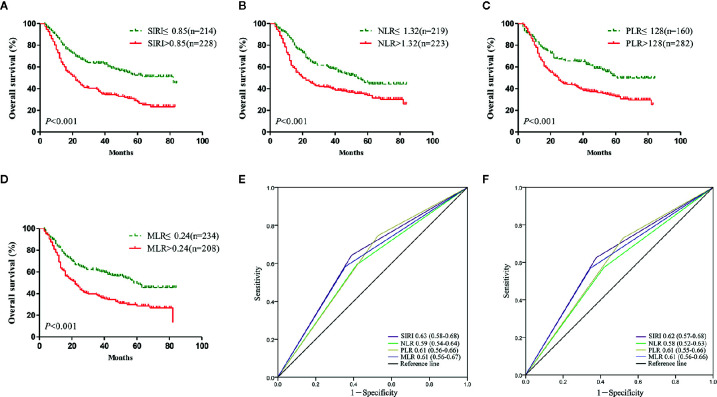
The prognostic significance of the systemic inflammation response index (SIRI) **(A)**, neutrophil/lymphocyte ratio (NLR) **(B)**, platelet/lymphocyte ratio (PLR) **(C)**, and monocyte/lymphocyte ratio (MLR) **(D)** in gastric cancer in the primary cohort. Predictive ability of the SIRI in gastric cancer was compared with PLR, NLR and MLR by receiver operating characteristic (ROC) curves in 3 years **(E)** and 5 years **(F)** in the primary cohort.

**Figure 3:**
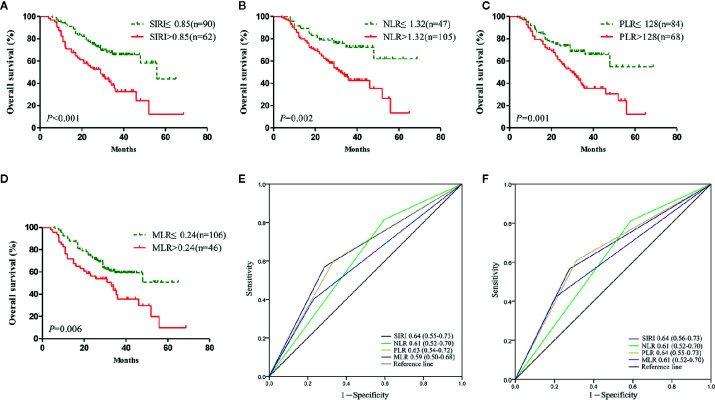
The prognostic significance of the systemic inflammation response index SIRI **(A)**, neutrophil/lymphocyte ratio (NLR) **(B)**, platelet/lymphocyte ratio (PLR) **(C)**, and monocyte/lymphocyte ratio (MLR) **(D)** in gastric cancer in the validation cohort. Predictive ability of the SIRI in gastric cancer was compared with PLR, NLR, and MLR by receiver operating characteristic (ROC) curves in 3 years **(E)** and 5 years **(F)** in the validation cohort.

Survival analysis and the log-rank test were used to analyze the relationships between SIRI, NLR, PLR, MLR, and the postoperative survival time of patients. In the primary cohort, Kaplan-Meier survival curves showed that the median OS of gastric cancer patients with SIRI ≤0.85 was 65 months, which was significantly longer than that of patients with SIRI >0.85 (22 months) (*P*<0.001) (**Figure 2A**). The OS of patients with NLR ≤1.32, PLR ≤128, and MLR ≤0.24 was significantly higher than that of patients with NLR>1.32, PLR>128, and MLR>0.24 (**Figures 2B–D**). Compared with NLR, PLR, and MLR, the area under the curve (AUC) of SIRI for the 3- and 5-year survival rates of gastric cancer patients was larger, indicating that SIRI has a better prognostic value for gastric cancer patients than the other systemic inflammation indexes (**Figures 2E, F**). In the validation cohort, patients with gastric cancer with SIRI ≤0.85, NLR ≤1.32, PLR ≤128, and MLR ≤0.24 also had significantly longer OS times than those with SIRI >0.85, NLR >1.32, PLR >128, and MLR >0.24 (**Figures 3A–D**). The AUC of SIRI before surgery was also larger than that of the other systemic inflammatory indexes (**Figures 3E, F**). The prognostic value of SIRI before surgery was confirmed in two different cohorts, and its value for judging the prognosis was greater than that of NLR, PLR, and MLR.

Univariate analysis using Cox regression models confirmed the clinicopathological parameters that were useful for predicting the clinical prognosis. In the primary cohort, univariate analysis found that the tumor location, grade, tumor size, perineural invasion, TNM stage, and systemic inflammation indexes were significant prognostic factors for affecting gastric cancer patients. Subsequent multivariate analysis showed that grade, TNM stage, and SIRI were independent risk factors for OS (**Table 3**). The results of the validation cohort were basically similar, but it was found that grade was not a factor correlated with the prognosis of gastric cancer patients (**Table 3**).

**Table 3:** Univariate and multivariate cox regression analyses for overall survival in patients with gastric cancer.

Variables	Univariate analysis		Multivariate analysis	
	HR (95% CI)	*P* value	HR (95% CI)	*P* value
**Primary cohort**				
Sex: male *vs*. female	1.00 (0.77–1.29)	0.976		
Age: >60 *vs*. ≤60	1.23 (0.94–1.61)	0.136		
Tumor location		0.004^*^		0.087
Middle *vs*. upper	0.73 (0.52–1.02)	0.063	0.80 (0.56–1.13)	0.201
Lower *vs*. upper	0.57 (0.40–0.79)	0.001^*^	0.66 (0.46–0.96)	0.028^*^
Grade: poorly *vs*. well	2.20 (1.71–2.83)	<0.001^*^	1.42 (1.07–1.87)	0.014^*^
Lauren type		0.843		
Intestinal *vs*. diffuse	1.05 (0.74–1.49)	0.797		
Mixed *vs*. diffuse	0.97 (0.67–1.39)	0.853		
Tumor size: >5 *vs*. ≤5	2.04 (1.57–2.67)	<0.001^*^	1.26 (0.94–1.70)	0.127
Lymphovascular: yes *vs*. no	1.17 (0.89–1.54)	0.256		
Perineural: yes *vs*. no	1.74 (1.36–2.23)	<0.001^*^	1.18 (0.89–1.56)	0.247
TNM stage		<0.001^*^		<0.001^*^
II *vs*. I	2.32 (1.45–3.72)	<0.001^*^	1.90 (1.17–3.07)	0.009^*^
III *vs*. I	6.17 (4.00–9.48)	<0.001^*^	3.89 (2.47–6.14)	<0.001^*^
Chemotherapy: Yes *vs*. no	1.15 (0.90–1.48)	0.282		
SIRI: >0.85 *vs*. ≤0.85	2.06 (1.60–2.66)	<0.001^*^	1.47 (1.11–1.94)	0.006^*^
NLR: >1.32 *vs*. ≤1.32	1.69 (1.31–2.16)	<0.001^*^	1.23 (0.90–1.71)	0.196
PLR: >128 *vs*. ≤128	1.83 (1.39–2.42)	<0.001^*^	1.19 (0.88–1.59)	0.256
MLR: >0.24 *vs*. ≤0.24	1.86 (1.45–2.39)	<0.001^*^	1.27 (0.98–1.66)	0.073
Dynamic change in SIRI		<0.001^*^		<0.001*
SIRI no change *vs*. decrease>50%	2.33 (1.57–3.47)	<0.001^*^	1.90 (1.27–2.86)	0.002^*^
increase>50% *vs*. decrease>50%	4.52 (2.89–7.05)	<0.001^*^	4.01 (2.55–6.30)	<0.001^*^
**Validation cohort**				
Sex: male *vs*. female	0.98 (0.56–1.69)	0.932		
Age: >60 *vs*. ≤60	1.21 (0.94–1.57)	0.141		
Tumor location		0.833		
Middle *vs*. upper	0.78 (0.35–1.75)	0.547		
Lower *vs*. upper	0.80 (0.34–1.88)	0.614		
Grade: poorly *vs*. well	1.27 (0.99–1.61)	0.051		
Lauren type		0.795		
Intestinal *vs*. diffuse	1.21 (0.62–2.37)	0.574		
Mixed *vs*. diffuse	1.26 (0.64–2.49)	0.507		
Tumor size: >5 *vs*. ≤5	2.07 (1.21–3.55)	0.008^*^	1.27 (0.56–2.89)	0.570
Lymphovascular: yes *vs*. no	1.43 (0.86–2.36)	0.168		
Perineural: yes *vs*. no	1.85 (1.35–2.55)	<0.001^*^	1.63 (0.71–3.74)	0.250
TNM stage		<0.001^*^		<0.001^*^
II *vs*. I	2.55 (1.26–5.19)	0.010^*^	1.99 (0.93–4.25)	0.075
III *vs*. I	6.02 (2.87–12.63)	<0.001^*^	4.95 (2.14–11.47)	<0.001^*^
Chemotherapy: yes *vs*. no	1.42 (0.88–2.31)	0.154		
SIRI: >0.85 *vs*. ≤0.85	2.49 (1.54–4.01)	<0.001^*^	2.21 (1.18–4.15)	0.001^*^
NLR: >1.32 *vs*. ≤1.32	2.49 (1.36–4.58)	0.003^*^	1.80 (0.91–3.56)	0.154
PLR: >128 *vs*. ≤128	2.15 (1.32–3.49)	0.002^*^	1.56 (0.87–2.80)	0.132
MLR: >0.24 *vs*. ≤0.24	1.94 (1.20–3.13)	0.007^*^	1.62 (0.98–2.69)	0.060
Dynamic change in SIRI		<0.001^*^		<0.001*
SIRI no change *vs*. decrease>50%	2.70 (1.23–5.89)	0.013^*^	2.18 (0.96–4.95)	0.064
Increase>50% *vs*. decrease>50%	6.54 (3.14–13.63)	<0.001^*^	4.93 (2.28–10.67)	<0.001^*^

*represents statistical significance.

### Prognostic Value of Dynamic Changes in SIRI

**Figure 4:**
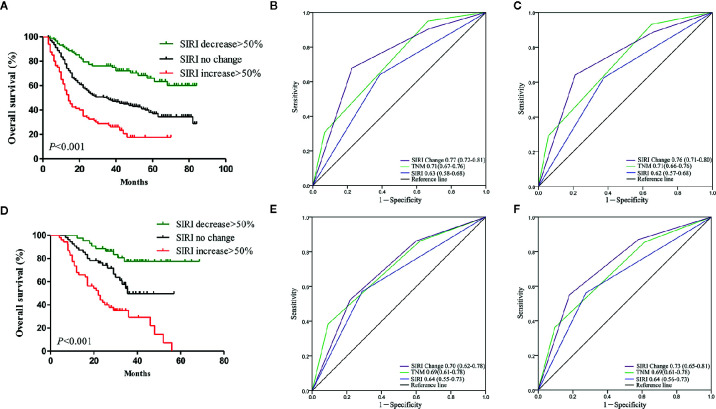
**(A)** Dynamic changes of systemic inflammation response index (SIRI) before and after radical gastrectomy had significant effects on survival in the primary cohort. Predictive ability of SIRI, dynamic changes of SIRI was compared with TNM stage by receiver operating characteristic (ROC) curves in 3 years **(B)** and 5 years **(C)** in the primary cohort. **(D)** Dynamic changes of SIRI before and after radical gastrectomy had significant effects on survival in the validation cohort. Predictive ability of SIRI, dynamic changes of SIRI was compared with TNM stage by ROC curves in 3 years **(E)** and 5 years **(F)** in the validation cohort.

The dynamic change in SIRI was calculated as described above and then divided into three groups [decrease>50%, no change (decrease or increase of no more than 50%), and increase>50%]. We retrospectively analyzed whether the dynamic change of the SIRI was related to adjuvant therapy. In the primary cohort, 163/274 patients with SIRI no change, 53/88 patients with SIRI decrease>50%, and 49/80 patients with SIRI increase>50% received chemotherapy. In the verification cohort, there were 38/55, 21/44, and 36/53 patients who received chemotherapy in the SIRI no change group, SIRI decrease>50% group, and SIRI increase>50% group. The dynamic change of the SIRI had no correlation with the proportion of adjuvant chemotherapy, whether in the primary cohort or the verification cohort. In the primary cohort, the Kaplan-Meier survival curve showed that the OS of patients with SIRI decrease >50% was significantly higher than that of patients with no change in SIRI (*P*<0.001), while patients with SIRI increase >50% had significantly lower OS rates than patients with no change in SIRI (*P*<0.001) (**Figure 4A**). Multivariate analysis showed that dynamic change in SIRI is an independent prognostic factor for gastric cancer (**Table 3**). Then, the AUC was used to compare the prognostic value of SIRI and TNM stage. The results showed that the 3- and 5-year survival rates of gastric cancer patients were predicted successfully. The AUC of the SIRI change was larger than that of the SIRI and the TNM stage, suggesting that the change in SIRI has a better prognostic value for patients with gastric cancer than SIRI and TNM stage (**Figures 4B, C**). In the validation cohort, the same results were obtained (**Figures 4D–F**). The two cohorts also confirmed that the dynamic changes in SIRI could be used to evaluate the prognosis of operable gastric cancer, and the prognostic value was not less than that of the TNM stage.

## Discussion

In recent years, tumor-related inflammation has become one of the new hot spots in tumor research (14). The inflammatory state of the immune system microenvironment and its changes are related to tumor development (3). Previous studies have found that some systemic immune response indexes, such as CRP, NLR, the Glasgow Prognosis Score (GPS), and PLR, can predict the clinical outcomes of gastric cancer patients (5–8). A number of recent studies have shown the prognostic value of SIRI in a variety of cancers (12, 13, 15). Li and Zhang separately confirmed that SIRI is a useful prognostic index for identifying gastric cancer patients with a poor prognosis, and the prognosis of patients with a high SIRI is poor (10, 11). In this study, we independently evaluated the prognostic value of the preoperative SIRI, NLR, PLR, and MLR in patients undergoing radical gastrectomy in two centers and found that SIRI is an independent prognostic factor for the OS of patients undergoing radical gastrectomy, and the ability of SIRI to predict patient outcomes is superior to that of the other inflammatory indexes. In addition, the dynamic changes in SIRI before and after surgery can also be used to evaluate the prognosis of patients with gastric cancer, and its the prediction ability is better than that of TNM stage. This is the first report of the prognostic value of the dynamic changes in SIRI before and after surgery in gastric cancer. SIRI have the advantages of high accessibility, low cost and reproducibility. Similar to the classical tumor markers, it can change with the changes of tumor load and immune response status in patients. The dynamic changes could accurately reflect the trend of tumor progression and response to therapy. In addition, SIRI was able to reflect the status of the local immune response and systemic inflammation. Therefore, SIRI may serve as an “immunologic signature” in gastric cancer and potentially could be used to predict response to immunotherapy, which would allow clinicians to tailor future therapies for those patients who would benefit most from immunotherapy (9).

Studies have revealed that increased numbers of neutrophils can secrete a large amount of reactive oxygen species, induce cellular DNA damage and trigger genetic instability, causing tumorigenesis and promoting tumor progression in the tumor microenvironment (16). Increased levels of circulating neutrophils secrete angiogenic cytokines, such as VEGF, which is beneficial for tumor angiogenesis (17). As an important part of the body’s immune response function, lymphocyte populations play an important role in antitumor immunity. In the process of tumorigenesis, lymphocytes in the peripheral blood can migrate to the tumor microenvironment, form TILs, and exert antitumor effects (18). Among them, CD8+ cytotoxic lymphocytes (CTLs) are considered to be the main antitumor immune effector cells. They can recognize tumor antigens and directly cause tumor cell death. When activated, these cells can produce cytotoxins such as perforin and granzyme to kill tumor cells or induce tumor cell apoptosis through the Fas-FasL pathway. In addition to CD8+ T cells, CD4+ helper T cells also play an important role in the body’s antitumor immunity. In recent years, the role of monocytes and their differentiated macrophages in tumorigenesis and progression has gradually attracted the attention of researchers, but the specific mechanism has not yet been fully elucidated. Peripheral blood monocytes chemotactically migrate to the tumor microenvironment and may promote tumor progression through the following mechanisms. (1) These tumor-acclimated monocytes upregulate the expression levels of CCL2, CCL17, CCL24, CXCL6, and other chemokines and recruit these chemokines, myeloid-derived inhibitory cells and regulatory T cells, thereby causing negative immune regulation (19). (2) Monocytes enhance the function of IL-6 and GM-CSF and other tumor-promoting cytokines (20). (3) Monocytes can express PD-L1, CTLA4, and other common inhibitors at high levels, causing strong immunosuppression in the tumor microenvironment (21). (4) Monocytes secrete matrix metalloproteinases such as MMP-2 and MMP-9 and accelerate tumor metastasis by regulating epithelial proliferation and angiogenesis (22). Since SIRI is calculated based on peripheral neutrophil, monocyte and lymphocyte counts, we can only speculate from the possible outcomes of these three cells disorder. SIRI is a more objective marker for the balance of the host inflammation and immune response. It reflected the complex interactions and potentially synergistic effects among neutrophils, monocytes/macrophages, and lymphocytes in the tumor microenvironment. An increase in SIRI may represent something happening at the pathophysiologic level with micrometastatic disease/cancer stem cells, or it may represent a change in the patient’s immune function after surgery. Therefore, we need further research to explore the underlying mechanism that increased SIRI can be used to predict the recurrence of gastric cancer.

It is undeniable that our study has certain limitations. (1) This study is a retrospective analysis, and future prospective, large sample studies are needed to verify the prognostic value of SIRI in gastric cancer and determine the best SIRI cut-off value. (2) This study did not test a variety of inflammatory cells, such as TILs and TAMs, in tumor tissue samples. Whether the peripheral blood cell count can accurately reflect inflammation and immune status in the tumor microenvironment is unknown. (3) The related mechanisms of the dynamic change in SIRI and the prognostic prediction of gastric cancer are still unclear and need to be studied and confirmed in depth. (4) We have tried to use disease free survival (DFS) as an indicator of prognosis. However, the patients included in this study came from different hospitals, and the frequency of postoperative follow-up was different, and some were even irregular, resulting in inaccurate recurrence time: inaccurate DFS will affect the accuracy of the results. So DFS cannot be used as outcome measure in this study. Despite these limitations, we believe that the results of this study c support the prognostic value of SIRI in gastric cancer.

In general, SIRI can be used as a reliable index to evaluate the prognosis of patients with operable gastric cancer, and the dynamic changes in SIRI before and after surgery are significantly related to the prognosis of patients with gastric cancer. As a non-invasive, low-cost, simple, and repeatable index, the SIRI can more objectively and reliably predict the survival of gastric cancer patients after a radical operation and help clinicians stratify the risk of death of gastric cancer patients and formulate reasonable individualized treatment plans.

## Data Availability Statement

The raw data supporting the conclusions of this article will be made available by the authors, without undue reservation.

## Ethics Statement

The studies involving human participants were reviewed and approved by Yancheng City No. 1 People’s Hospital and Yancheng Third People’s Hospital. The patients/participants provided their written informed consent to participate in this study.

## Author Contributions

ZL and HS conceived and designed the study and helped to draft the manuscript. ZL and HG performed the data collection. ZM and SS performed the statistical analysis. CD revised the manuscript. All authors contributed to the article and approved the submitted version.

## Conflict of Interest

The authors declare that the research was conducted in the absence of any commercial or financial relationships that could be construed as a potential conflict of interest.

## References

[1] Bray F Ferlay J Soerjomataram I Siegel RL Torre LA Jemal A. Global cancer statistics 2018: GLOBOCAN estimates of incidence and mortality worldwide for 36 cancers in 185 countries. CA Cancer J Clin (2018) 68:394–424. 10.3322/caac.21492 30207593

[2] Chen W Zheng R Baade PD Zhang S Zeng H Bray F. Cancer statistics in China, 2015. CA Cancer J Clin (2016) 66:115–32. 10.3322/caac.21338 26808342

[3] Mantovani A Allavena P Sica A Balkwill F. Cancer-related inflammation. Nature (2008) 454:436–44. 10.1038/nature07205 18650914

[4] Lee K Hwang H Nam KT. Immune response and the tumor microenvironment: how they communicate to regulate gastric cancer. Gut Liver (2014) 8:131–9. 10.5009/gnl.2014.8.2.131 PMC3964262 24672653

[5] Jung MR Park YK Jeong O Seon JW Ryu SY Kim DY. Elevated preoperative neutrophil to lymphocyte ratio predicts poor survival following resection in late stage gastric cancer. J Surg Oncol (2011) 104:504–10. 10.1002/jso.21986 21618251

[6] Shi H Jiang Y Cao H Zhu H Chen B Ji W. Nomogram Based on Systemic Immune-Inflammation Index to Predict Overall Survival in Gastric Cancer Patients. Dis Markers (2018) 2018):1787424. 10.1155/2018/1787424 30627220 PMC6305021

[7] Pan YC Jia ZF Cao DH Wu YH Jiang J Wen SM. Preoperative lymphocyte-to-monocyte ratio (LMR) could independently predict overall survival of resectable gastric cancer patients. Med (Baltimore) (2018) 97:e13896. 10.1097/MD.0000000000013896 PMC6314713 30593200

[8] Saito H Kono Y Murakami Y Shishido Y Kuroda H Matsunaga T. Prognostic Significance of Platelet-Based Inflammatory Indicators in Patients with Gastric Cancer. World J Surg (2018) 42:2542–50. 10.1007/s00268-018-4527-8 29464343

[9] Qi Q Zhuang L Shen Y Geng Y Yu S Chen H. A novel systemic inflammation response index (SIRI) for predicting the survival of patients with pancreatic cancer after chemotherapy. Cancer (2016) 122:2158–67. 10.1002/cncr.30057 27152949

[10] Li S Lan X Gao H Li Z Chen L Wang W. Systemic Inflammation Response Index (SIRI), cancer stem cells and survival of localised gastric adenocarcinoma after curative resection. J Cancer Res Clin Oncol (2017) 143:2455–68. 10.1007/s00432-017-2506-3 PMC11819166 28828692

[11] Zhang J Ding Y Wang W Lu Y Wang H Teng L. Combining the Fibrinogen/Albumin Ratio and Systemic Inflammation Response Index Predicts Survival in Resectable Gastric Cancer. Gastroenterol Res Pract (2020) 2020:3207345. 10.1155/2020/3207345 32184816 PMC7060846

[12] Geng Y Zhu D Wu C Wu J Wang Q Li R. A novel systemic inflammation response index (SIRI) for predicting postoperative survival of patients with esophageal squamous cell carcinoma. Int Immunopharmacol (2018) 65:503–10. 10.1016/j.intimp.2018.10.002 30408627

[13] Chao B Ju X Zhang L Xu X Zhao Y. A Novel Prognostic Marker Systemic Inflammation Response Index (SIRI) for Operable Cervical Cancer Patients. Front Oncol (2020) 10:766. 10.3389/fonc.2020.00766 32477958 PMC7237698

[14] Hanahan D Weinberg RA. Hallmarks of cancer: the next generation. Cell (2011) 144:646–74. 10.1016/j.cell.2011.02.013 21376230

[15] Chen Y Jin M Shao Y Xu G. Prognostic Value of the Systemic Inflammation Response Index in Patients with Adenocarcinoma of the Oesophagogastric Junction: A Propensity Score-Matched Analysis. Dis Markers (2019) 2019):4659048. 10.1155/2019/4659048 31781301 PMC6875417

[16] Weitzman SA Gordon LI. Inflammation and cancer: role of phagocyte-generated oxidants in carcinogenesis. Blood (1990) 76:655–63. 10.1182/blood.V76.4.655.655 2200535

[17] Shamamian P Schwartz JD Pocock BJ Monea S Whiting D Marcus SG. Activation of progelatinase A (MMP-2) by neutrophil elastase, cathepsin G, and proteinase-3: a role for inflammatory cells in tumor invasion and angiogenesis. J Cell Physiol (2001) 189:197–206. 10.1002/jcp.10014 11598905

[18] Ray-Coquard I Cropet C Van Glabbeke M Sebban C Le Cesne A Judson I. Lymphopenia as a prognostic factor for overall survival in advanced carcinomas, sarcomas, and lymphomas. Cancer Res (2009) 69:5383–91. 10.1158/0008-5472.CAN-08-3845 PMC2775079 19549917

[19] Gao Q Zhao YJ Wang XY Qiu SJ Shi YH Sun J. CXCR6 upregulation contributes to a proinflammatory tumor microenvironment that drives metastasis and poor patient outcomes in hepatocellular carcinoma. Cancer Res (2012) 72:3546–56. 10.1158/0008-5472.CAN-11-4032 22710437

[20] Bingle L Brown NJ Lewis CE. The role of tumour-associated macrophages in tumour progression: implications for new anticancer therapies. J Pathol (2002) 196:254–65. 10.1002/path.1027 11857487

[21] Liu LZ Zhang Z Zheng BH Shi Y Duan M Ma LJ. CCL15 Recruits Suppressive Monocytes to Facilitate Immune Escape and Disease Progression in Hepatocellular Carcinoma. Hepatology (2019) 69:143–59. 10.1002/hep.30134 30070719

[22] Mantovani A Allavena P Sica A. Tumour-associated macrophages as a prototypic type II polarised phagocyte population: role in tumour progression. Eur J Cancer (2004) 40:1660–7. 10.1016/j.ejca.2004.03.016 15251154

